# Nidulantes of Aspergillus (Formerly Emericella): A Treasure Trove of Chemical Diversity and Biological Activities

**DOI:** 10.3390/metabo10020073

**Published:** 2020-02-17

**Authors:** Najla Ali Alburae, Afrah E. Mohammed, Hajer Saeed Alorfi, Adnan Jaman Turki, Hani Zakaria Asfour, Walied Mohamed Alarif, Ahmed Abdel-Lateff

**Affiliations:** 1 Department of Biology, Faculty of Science, King Abdulaziz University, P.O. Box 80203, Jeddah 21589, Saudi Arabia; nalbourai@kau.edu.sa; 2 Department of Biology, Faculty of Science, Princess Nourah bint Abdulrahman University, P.O. Box 84428, Riyadh 11671, Saudi Arabia; AFAMohammed@pnu.edu.sa; 3 Department of Chemistry, Faculty of Science, King Abdulaziz University, P.O. Box 80203, Jeddah 21589, Saudi Arabia; halorfi@kau.edu.sa; 4 Department of Marine Chemistry, Faculty of Marine Sciences, King Abdulaziz University, P.O. Box 80207, Jeddah 21589, Saudi Arabia; aturki@kau.edu.sa; 5 Department of Medical Microbiology and Parasitology, Faculty of Medicine, Princess Al-Jawhara Center of Excellence in Research of Hereditary Disorders, King Abdulaziz University, Jeddah 21589, Saudi Arabia; hasfour@kau.edu.sa; 6 Department of Natural Products and Alternative Medicine, Faculty of Pharmacy, King Abdulaziz University, P.O. Box 80260, Jeddah 21589, Saudi Arabia; 7 Department of Pharmacognosy, Faculty of Pharmacy, Minia University, Minia 61519, Egypt

**Keywords:** ascomycota, fungi, polyketides, alkaloids, cyclic peptides, antimicrobial, anti-oxidant, anti-inflammatory

## Abstract

The genus *Emericella* (Ascomycota) includes more than thirty species with worldwide distribution across many ecosystems. It is considered a rich source of diverse metabolites. The published classes of natural compounds that are discussed here are organized according to the following biosynthetic pathways: polyketides (azaphilones, cyclopentenone pigments, dicyanides, furan derivatives, phenolic ethers, and xanthones and anthraquinones); shikimate derivatives (bicoumarins); mevalonate derivatives (meroterpenes, sesquiterpenes, sesterterpenes and steroids) and amino acids derivatives (alkaloids (indole-derivatives, isoindolones, and piperazine) and peptides (depsipeptides)). These metabolites produce the wide array of biological effects associated with *Emericella*, including antioxidant, antiproliferative, antimalarial, antiviral, antibacterial, antioxidant, antihypertensive, anti-inflammatory, antifungal and kinase inhibitors. Careful and extensive study of the diversity and distribution of metabolites produced by the genus *Emericella* (either marine or terrestrial) revealed that, no matter the source of the fungus, the composition of the culture medium effectively controls the metabolites produced. The topic of this review is the diversity of metabolites that have been identified from *Emericella*, along with the contextual information on either their biological or geographic sources. This review presents 236 natural compounds, which were reported from marine and terrestrial *Emericella*. Amongst the reported compounds, only 70.2% were biologically assayed for their effects, including antimicrobial or cytotoxicity. This implies the need for substantial investigation of alternative activities. This review includes a full discussion of compound structures and disease management, based on materials published from 1982 through December 2019.

## 1. Introduction

Fungi are ubiquitous cosmopolitan organisms, accounting for the second largest group of organisms after insects [1]. Inter alia, until the discovery of antibiotics, fungi have been known for decades as harmful entities, spoiling foods and causing health hazards; today they are considered as antimicrobials that act against bacteria, which has been saving millions of lives. Views on fungi have changed to consider their beneficial effects, which have appeared in bread preparation, cheese manufacturing and fermentation [1,2,3,4]. The number of fungal strains is roughly estimated to be six per plant species. Consequently, the total number of fungi is tentatively estimated as 1.5 million species, of which only 100,000 species have been identified [4]. The classified fungal species population on earth is tentatively estimated to be 150,000, which indicates that only 10% have been scientifically investigated [5,6]. Kirk et al. (2008) deduced that the fungal population is growing at a rate of 1200 species per year [7]. Consequently, more time is required to describe the remaining fungal species. Fungi include marine and terrestrial, endophytic and saprophytic and pathological and mutualistic species [8]. 

Endophytic fungi spend part or all of their lives colonizing the tissues of their hosts [2,3]. They can be categorized into obligate or facultative. The fungi that depend on taking nutrients from their surroundings are termed as obligate [8,9]. Conversely, facultative fungi live outside the host body during a definite stage of their lives and are mostly associated with plants from the neighboring soil environment and atmosphere [10]. Their relationships range from symbiotic to pathogenic. The host organisms supply the nutrients to the endophytic microorganisms, and in return, they produce secondary products as growth and competitiveness factors for the host in its environment [11]. Recently, they have been identified as a fruitful scaffold for bioactive natural compounds, thus demonstrating possible leads for new phytopharmacological agents [10,12]. Marine fungi produce interesting metabolites, which has attracted particular attention among those seeking new pharmaceuticals [13]. The marine sediment offers a wealth of secondary metabolites with exceptional structures and interesting biological effects [14].

Natural products represent eighty percent of all pharmaceuticals. For example, of the seventy thousand reported compounds of polyketide origin, more than twenty developed substances are now available in medical markets. This hit rate of less than one percent is notably higher than that for screening of synthetic pharmaceutical agents, which is less than 0.001% [15]. Researchers have considered such fungal applications since the discovery of penicillin and the trend has only increased with time. Currently, more than thirty-five launched pharmaceutics were originally discovered from fungi [16]. These drugs were employed to manage certain diseases including bacterial and fungal infections, to serve as immunosuppressants, and as other therapeutic agents. Those that have been processed in clinical studies include plinabulin and diketopiperazine derivatives [17]. It is well-known that the antimicrobial agent cephalosporin C, which was originally discovered in July 1945, was developed from the marine fungus *Acremonium strictum W. Gams* (1971) [18]. Despite these momentous discoveries, however, the number of active marine fungal products has increased only slowly. An interesting method of engaging the fungal warehouse is the One Strain-Many Compounds Approach (OSMAC), which includes managing the culture conditions such as media composition, temperature pH and oxygen source [19,20,21,22]. 

**Figure 1:**
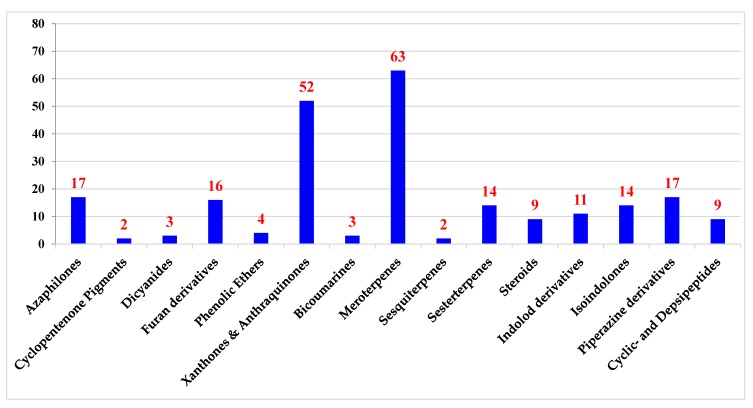
Diversity of chemical classes obtained from the genus *Emericella*.

Genus *Emericella* (*E.*) includes more than thirty species and is named after the sexual phase (teleomorph) of this fungal strain, which ranges from saprobes to pathogenic or endophytic organisms on living host organism [23]. It forms white cleistothecia surrounded by Hülle cells and produces purple ascospores. Conidiophores usually have short brown stripes, bear both metulae and phialides and produce columns of dark green conidia [24,25]. The white cleistothecia surrounded by Hülle cells are characteristic of *Emericella*. A published taxonomical study of *Emericella* species resulted in transferring them to a subgenus of *Aspergillus* named *"Nidulantes"*. The subgenus *Nidulantes* consists of five sections (*Nidulantes, Versicolores, Usti, Terrei* and *Flavipedes*). This is based on the fact that the majority of the *Emericella* have a sexual stage, and that their morphological features are similar to those of *Aspergillus*. *Nidulantes* species are able to produce a sexual state and those species were, in the dual name nomenclature system, assigned to the genus *Emericella*. Because of the adoption of the “one fungus: one name” nomenclatural system, the majority of *Emericella* species have been transferred to *Aspergillus*. A summary of a reported study interested in extrolites obtained from different sections of subgenus *Nidulantes* indicated that the reported metabolites are more or less similar to those produced from other sections. For instance, sterigmatocystins, shamixanthones and violaceols are noticeable in many species [26]. Herein, the metabolites’ chemical structure and biological effects reported from genus *Emericella* are discussed, whether derived from marine (51 metabolites) or terrestrial (185 metabolites) environments. *Emericella* is considered a rich source for discovering new pharmaceutical agents with a remarkable diversity of molecular structures. The published classes of natural compounds that are discussed here are organized according to the biosynthetic pathways polyketides (azaphilones, cyclopentenone pigments, dicyanides, furan derivatives, phenolic ethers, xanthones and anthraquinones); Shikimate derivatives (bicoumarins); Mevalonate derivatives (meroterpenes, sesquiterpenes, sesterterpenes and steroids) and amino acids derivatives (alkaloids (indole-derivatives, isoindolones, and piperazine) and peptides (depsipeptides)) (Figure 1). A full discussion of 236 metabolites, which have been published between 1982 and December 2019, is presented. All the information about *Emericella* was obtained through searching journals, books and electronic databases, including Web of Science, SciFinder, Science Direct, PubMed, Elsevier, Google Scholar, Wiley, American Chemical Society and Springer. 

## 2. Polyketides 

### 2.1. Azaphilones 

Azaphilones (isochromenes) are fungal metabolites with a pyrone-quinone skeleton and a highly oxygenated bicyclic structure. They have the ability to react with amines to form red or purple vinylogous c-pyridones due to the exchange of pyrane oxygen for nitrogen. This class of natural substances is responsible for the characteristic colors of the mycelia. Azaphilones play an important role as a taxonomically important marker in some fungal species. For instance, sassafrins were firstly found in *Creosphaeria sassafras*, and were fortunately not produced by any other species of the Xylariaceae [27]. Azaphilones are products of many species that belong to the basidiomycetous and ascomycetous species. They exhibit a variety of pharmacological effects, including antimicrobial, antiviral, cytotoxic, anticancer and anti-inflammatory effects. Several biological effects have been assigned to the reactions of azaphilones with the N-containing moieties, like proteins, nucleic acids and amino acids, resulting in the formation of vinylogous γ-pyridones [28]. 

**Figure 2:**
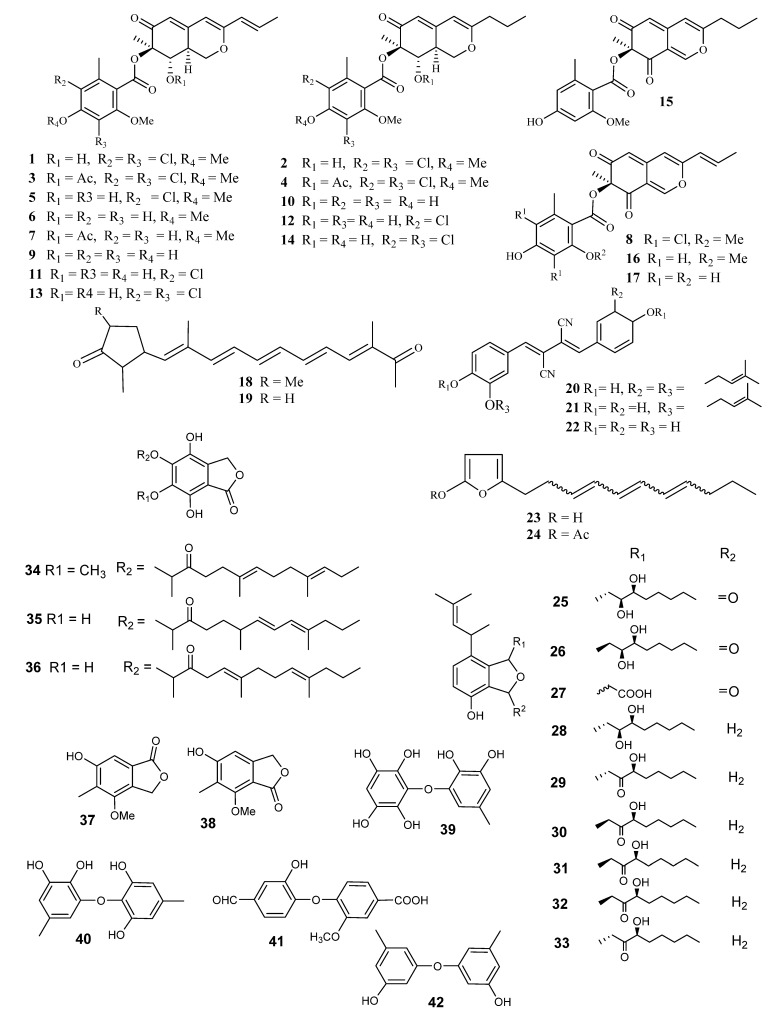
Structures of compounds **1–42.**

Four azaphilones, namely falconensins A–D (**1**–**4**) (Figure 2), were reported from Venezuelan soil *E. falconensis* (NHL 2999 = ATCC 76117). The dichloromethane extract, which was prepared from the mycelia of the fungus in a Czapek medium supported by 0.2% yeast extract, showed no antimicrobial activity against *Bacillus subtilis* Cohn and *Trichophyton mentagrophytes* [29]. 

Further investigations on the same strain by the same research group led to the identification of three more falconensins, namely E–G (**5–7**) [30]. They then studied the absolute stereochemistry of falconensin A (**1**). Hydrogenation of **1** resulted in a hexahydro derivative, which upon methanolysis gave a ketodiol, which was elucidated by extensive interpretation of different spectroscopic measurements including 1D and 2D NMR. An advanced Mosher’s approach was employed to identify the absolute stereochemistry of ketodiol [31]. The Mosher’s reagent [α-methoxy-α-(trifluoromethyl) phenylacetic acid (MTPA)] is used in measuring the chirality of the secondary alcohols, by generating the magnetic anisotropy effect of the ring current, which is induced under the external magnetic field [31]. The MTPA esters of ketodiol were prepared and the NMR assignment between these (*R*)- and (*S*)-MTPA esters was estimated. The absolute stereochemistry at C-8 was assigned as an *S* configuration, which is consistent with the outcome application of the octant rule to ketodiol. Conclusively, falconensins A–D had the same configuration by the comparison of their circular dichroism (CD) data. A positive cotton effect indicates an *R*- configuration at C-7 [30,31]. Further investigation of the same strain by the same scientists led to the isolation of a new azaphilone derivative, falconensin H (**8**). The absolute configuration around C-7 has been decisively recognized by measuring the optical rotations and CD measurements. Unfortunately, falconensins A–H showed no effect [32]. 

Six more reduced azaphilones, falconensins I–N (**9–14**) (Figure 2), along with the three new azaphilones monomethyldihydromitorubrin (**15**), mitorubrin (**16**) and monomethylmitorubrin (**17**) compounds, have been identified from the dichloromethane mycelial extract of *E. falconensis* and *E. fruticulosa* [33]. The chemical structures of the isolated compounds (**9**–**16**) were elucidated by interpretation of the measured spectra of ^1^H and ^13^C NMR and MS. The relative stereochemistry was determined from NOE NMR while the absolute stereochemistry was obtained by measuring the CD spectrum. For instance, the absolute configuration of falconensin I (**9**) has been elucidated from the signs of the bathochromic Cotton effect of the CD spectrum [∆^e^: + 7.8 (364 nm)] [33].

### 2.2. Cyclopentenone Pigments

Two new yellow-colored compounds, falconensones A and B (**18**,**19**), were reported from the dichloromethane extract of *E. falconensis* (NHL 2999 = ATCC 76117), isolated from Venezuelan soil and/or *E. fruticulosa*, which have been isolated from Venezuelan soil. Compounds **18** and **19** have cyclopent-2-en-1-one connected through conjugated pentaene with acetate residue [34]. 

Falconensone A (**18**) displayed an apoptotic effect on HL60 leukemia cells, while **19** and the 4’-nor-methyl derivative of falconensone A exhibited less potency [35]. The synthetic products, *p*-bromophenylhydrazone and dioxime derivatives of **18**, displayed higher potency than the corresponding non-synthetic falconensones A and B [36]. The initiation of cell death programming by the falconensones is associated with their inhibition of cell growth. The synthetic derivatives of falconensone A (i.e., falconensone A dioxime and falconensone A *p*-bromophenylhydrazone) have potent inhibitory effects on HL60 cell growth. They displayed inhibitory effects much greater than that exhibited by falconensone A. Unfortunately, **19** showed no measurable effect on cell growth. The proliferation effect of falconensone A and its derivatives on HL60 cell growth was inhibited at 1 and 10 µM. The acute growth inhibition by falconensone A *p*-bromophenyl-hydrazine was 55% continuously from 54 to 149 h; however, falconensone A dioxime and falconensone A inhibited cell growth gradually, exhibiting 35% and 23% cell growth inhibition at 54 and 149 h, respectively. These reported results indicate that **18** and its derivatives had antiproliferative effects. Moreover, the percentage of viable cells was >94% under all conditions for four days. Thus, the inhibition of cell growth cannot be attributed to necrotic cell death [35,36]. 

Furthermore, **18**, its dioxime and **19** elevated the generation of intracellular ROS (reactive oxygen species), while the *p*-bromophenylhydrazone of **18** is inactive [37]. These effects recommend that only **18** and its derivatives (i.e., *p*-bromophenylhydrazone and dioxime) would be considered as apoptotic agents. The active generation of ROS in cells may be induced only by **18** and its dioxime derivative. It was also proposed that the Me-4’ in the cyclopentenone ring in **18** may be essential for the induction of apoptosis. Thus, **18** and its derivatives may be clinically useful in the management of some leukemia types. Retinoic acid (RA) is a physiological compound that induces HL60 cells to change into granulocyte-like cells and undergo apoptosis. Falconensones induce differentiation of HL60 cells into monocyte/macrophage-like cells, and those combinations of falconensone A and RA synergistically induced differentiation. The structural similarity between RA or fenretinide and the falconensones, particularly between 4-HPR and falconensone A *p*-bromophenylhydrazone, led researchers to test falconensones, which could induce apoptosis of HL60 cells [35,36,37]. Fifteen falconensins were evaluated for their inhibitory impact on the enhancement of ear edema in mouse ears by 12-*O*-tetradecanoyl-phorbol-13-acetate [38]. These azaphilonoidal derivatives displayed inhibitory effects comparable with indomethacin. All the tested compounds, except **17**, inhibited the effect of inflammation with inhibitory doses (ID_50_) of 0.373, 0.553, 0.417 and 0.908, for falconensins E, K, M and indomethacin, respectively [35,36,37,38]. 

### 2.3. 1,4-diphenyl-2,3-dicyano-1,3-butadienes (Dicyanides)

Extraction with dichloromethane from the Egyptian desert soil strain *E. purpurea* IFO 30849, cultivated on potato-dextrose (PD), led to the isolation of three new yellow pigments, epurpurins A–C (**20**–**22**) (Figure 2). They were listed as the second report of fungal metabolites as examples of 1,4-diphenyl-2,3-dicyano-1,3-butadiene derivatives [39]. The chemical structures of (**20**–**22**) were confirmed by analyzing the spectral data of 1D (^1^H, ^13^C) NMR, 2D (^1^H-^13^C COLOC and^1^H-^1^H COSY) using NMR and MS. Of particular interest is the determination of the stereochemistry around the double bond of **20**–**22**. It was elucidated by measuring the coupling constants of the nitrile group, which was found to be 13.7 to 15.3 Hz. The *J*-value between the nitrile carbon and the olefinic proton depends on the stereochemistry of the double bond in the trisubstituted alkenes, i.e., the (*E*)-isomer showed a *J*-value in range of 7.7–17.0 Hz, whereas the (*Z*)-isomer showed a *J*-value in range of 4.3–10 Hz. This led to establishing the olefinic protons and the nitrile carbons to be *trans*. The structures of epurpurins A and B were consequently confirmed to be (2Z,3Z)- 1,4-di[4-hydroxy-3-(3-methyl-2-butenyl) phenyl]-2,3-dicyano-l,3-butadiene (**20**) and (2*Z*, 3*Z*) -1- [4-hydroxy-3-(3-methyl-2-butenyl)phenyl]-4-(4-hydroxyphenyl)-2,3-dicyano-1,3-butadiene (**21**) [39]. 

### 2.4. Furan Derivatives

Two alkylated furan derivatives were reported from the endophytic fungus *Emericella* sp. XL029 (isolated from the leaves of *Panax notoginseng*), namely 5-(undeca-3,5,7-trien-1-yl) furan-2-ol (**23**) and 5-(undeca-3,5,7-trien-1-yl)furan-2-carbonate (**24**) [40]. The double-bond stereochemistry could not be estimated due to the overlapping of signals in the olefinic region. Compounds **23**–**24** (Figure 2) were assayed for their anti-agricultural pathogenic effects, employing seven fungal and thirteen bacterial strains. Compound **23** displayed considerable effects against all assessed fungi (*Fusarium (F.) oxysporum, F. tricinctum, Botryosphaeria dothidea, and Alternaria fragriae*), with minimum inhibitory concentration (MIC) values ranging between 3.1–25 μg/mL, whereas **24** displayed impacts on all investigated fungi, except *Rhizoctonia solani* and *F. oxysporum*, with MIC values ranging between 12.5–50 μg/mL. Additionally, compounds **23** and **24** showed substantial inhibitory influences toward eight of the thirteen investigated bacteria, with MIC of 6.3–50 μg/mL [40]. 

Investigation of *Emericella* sp. IFM57991 led to the identification of fourteen new metabolites: three isobenzofuranone derivatives, emefuranones A1, A2 and B (**25–27**) (Figure 2); six new isobenzofuran derivatives, emefuran A, B1, B2, C1, C2 and D (**28–33**) and three new farnesylated isobenzofuranone derivatives, farnesylemefuranones A–C (**34**–**36**) (Figure 2) [41]. The antimicrobial and cytotoxic effects of compounds **25**–**34** were evaluated. Compounds **28**–**31** showed weak inhibitions against *B. subtilis* (inhibition zones, 8.5 to 13 mm in 250 μg per disk) [41]. 

Two isobenzofuranone, silvaticol (**37**) and nidulol (**38**)**,** were isolated from the acidulated dichloromethane of the mycelia of *E. desertorum*, which was isolated from Egyptian desert soils [42].

### 2.5. Phenolic Ethers

*E. violacea* IFO 8106 displayed toxigenicity against experimental animals through the production of phenolic metabolites [43,44]. The ethyl acetate extract of the mycelia, which were grown on sterilized rice, exhibited a lethal effect on mice upon intraperitoneal injection (500 mg/kg). Violaceol I (**39**) and II (**40**) (Figure 2) were isolated by employing silica gel column chromatography, together with another non-toxic phenolic compound, which was identified as violaceic acid (**41**). The aldehydic moiety was identified based on the appearance of a singlet at δ_H_ 9.65 ppm in the ^1^H NMR spectrum and a doublet at δ_C_ 190.6 ppm in ^1^H single-frequency off-resonance decoupling (SFORD) mode. 3,3’-dihydroxy-5,5’-dimethyldiphenyl ether (**42**) was previously reported from *E. rugulosa* [43]. 

### 2.6. Xanthones and Anthraquinones

Xanthones are natural metabolites characterized by having dibenzo-γ-pyrone derivatives. They are typically polysubstituted and occur as either partial or fully aromatized derivatives. Xanthones may be divided into different subclasses based upon their structural features: xanthone monomers and dimers/heterodimers. The notably wide array of xanthones’ bio-activities could be attributed to the production of several chemical classes, such as fully aromatic-, dihydro-, tetrahydro- and hexahydro-xanthones resulting from the oxidation of the C-ring [45,46]. 

Sterigmatocystin (**43**) was reported from *E. striata* and *E. venezualensis*, and also was known as a precursor of aflatoxin B1 [47,48]. Sterigmatocystin is polyketide, which produced by numerous species, including *Emericella* and *Aspergillus*. Its pathway is obeyed by the normal polyketide-derived compounds through polyketide synthases. Moreover, the gene (pksST) encoding the sterigmatocystin polyketide synthase in *A. nidulans* has been cloned, sequenced, and characterized. 

**Figure 3:**
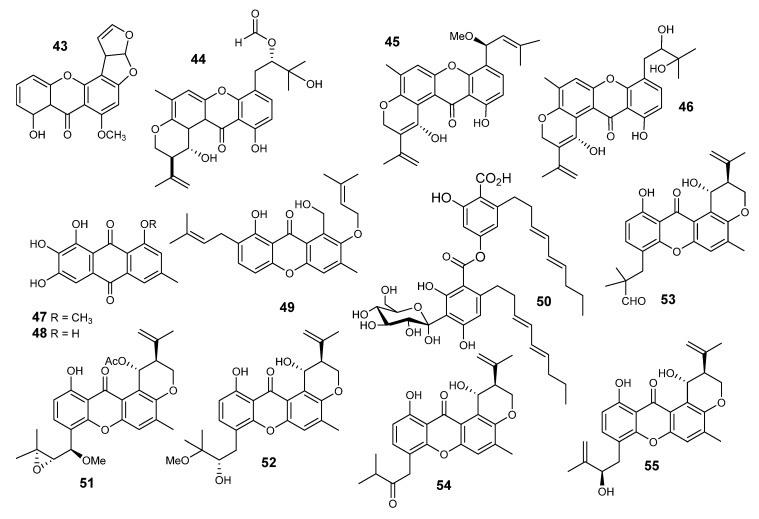
Structures of compounds **43–55**.

Varixanthone (**44**) (Figure 3), shamixanthone (**45**) and tajixanthone hydrate (**46**) were published from *E variecolor* [49,50,51]. Compound **45** displayed potent effects towards both Gram-positive and Gram-negative bacteria, as well as against the yeast *C. albicans*, higher than shamixanthone and tajixanthone hydrate. Varixanthone (**44**) was active with a value of MIC = 12.5 *µ*g/mL against *E. coli*, *Proteus* sp., *B. subtillis* and *S. aureus*. 

Investigation of marine *E. variecolor* led to identification of two new natural compounds. Evariquinone (**47**), a new anthraquinone known as 7-hydroxyemodin (**48**), new prenylxanthone isoemericellin (**49**) and shamixanthone (**45**) were identified [52]. Furthermore, the C-glycosidic depside stromemycin (**50**) (Figure 3) was reported, which has recently been patented for its metalloproteinase-inhibiting effect [53]. Evariquinone displayed an antiproliferative effect against KB and NCI-H460 cells (IC_50_ = 3.16 mg/ml). 

Four xanthones were reported from *E. variecolor* and were identified as shamixanthone (**45**), 14-methoxytajixanthone-25-acetate (**51**), tajixanthone methanoate (**52**) and tajixanthone hydrate (**46**). All of them showed moderate anti-tumor effects. Xanthones **45** and **46** have been shown to be as active as the standard doxorubicin hydrochloride toward gastric carcinoma (KATO3) and breast carcinoma (BT474) (Figure 3) [54].

**Figure 4:**
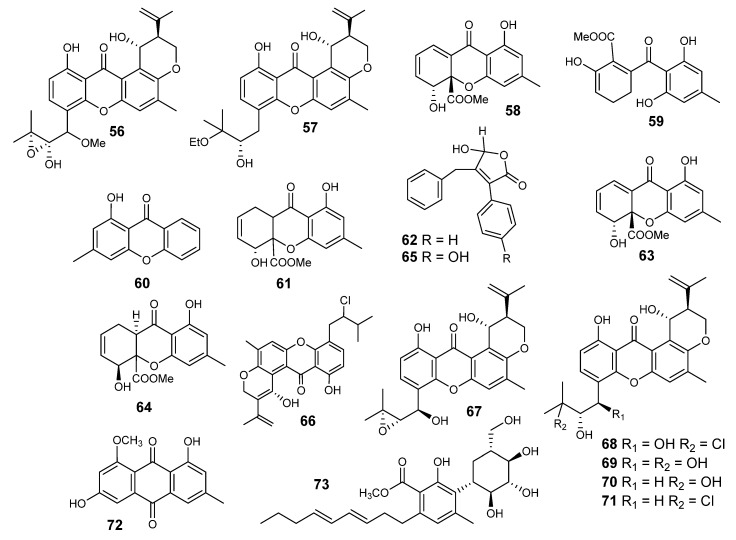
Structures of compounds **56–73**.

Ruguloxanthones A–C (**53–55**), 14-methoxytajixanthone (**56**) and tajixanthone ethanoate (**57**) (Figure 3 and Figure 4) along with the five known xanthones **43**, **46**, **47**, **50** and **52,** were reported from *E rugulosa* [55]. The organic extract, obtained from *E. quadrilineata* (IFM42027), suppressed the Con A-induced proliferation of mouse splenic lymphocytes by 96.6% at 50 µg/mL, but its treatment with *n*-hexane increased the activity by 3.1% [56]. Chromatographic separation of the defatted ethyl acetate extract led to the isolation of eight xanthone metabolites, identified as nidulalin A (**58**), nidulalin B (**59**), 1-hydroxy-3-methylxanthone (**60**), (4R,4aS,9aR)-1,9a-dihydronidulalin A (**61**), microperfuranone (**62**), pinselin (**63**), (4S,4aS,9aR)-4a-carbomethoxy-1,4,4a,9a-tetrahydro-4,8- dihydroxy-6-methyl xanthone (**64**) and 9-hydroxy microperfuranone (**65**) (Figure 4) [57]. The immunosuppressive activities of **46** and **52**–**62**, were estimated against Concanavalin A (Con A) induced (T-cell) and lipopolysaccharide (LPS)-induced proliferations of mouse splenic lymphocytes. Compound **58** possessed significant immunosuppressive effects, while **52** had a moderate effect. The fact that the immunosuppressive activities of **50** and **54** were less than that of **58** suggests that the presence of a C=C bond between position 1 and 9a in **53** might be important for the immunosuppressive effect. Compound **64** showed less activity than that of **57,** also suggesting that the presence of the free OH group at position 25 in **52** might be important for the appearance of the activity. It was already known that the suppressive effects of substituted xanthones against the proliferation of human lymphocytes were ascribable to the positions of substituents on the xanthone nucleus. Nidulalin A (**58**) was found to exhibit an inhibitory effect against DNA topoisomerases. This effect explains its cytotoxic mechanism (**53**) [57]. DNA topoisomerases are important enzymes that regulate the state of DNA topology, and thus they are important for DNA replication, recombination and transcription [57]. 

Investigation of *Emericella* sp. 25379 led to the identification of 15-chlorotajixanthone hydrate (**66**) and 14-methoxytajixanthone (**56**). In addition, shamixanthone (**45**) and tajixanthone hydrate (**46**) (Figure 4) were also obtained [58]. The activation of the calmodulin sensitive cAMP phosphodiesterase (PDE1) was inhibited by **45**, **46, 52** and **66** in a dose concentration-related manner [52]. Compounds **52** (IC_50_ = 5.54 µM) and **53** (IC_50_ = 5.62 µM) were comparable with chlorpromazine (IC_50_ = 7.26 µM, positive control), a well-known calmodulin (CaM) inhibitor. The modulation of physiological CaM targets by natural or synthetic compounds offers great possibilities for the discovery of new leads for the development of herbicide or therapeutically useful agents. Conclusively, AutoDock results indicated interactions of compounds **45**, **46, 52** and **66** with the protein at the same binding site of TFP, a well-known calmodulin (CaM) inhibitor. The CaM antagonist effect of **53** might be related with its mild cytotoxic action [58]. 

Four new prenylatedxanthones (**67**–**70**), along with three known compounds (**71**–**73**) (Figure 4), were isolated from *Emericella* sp. XL029 [59]. All seven pigments (**67**–**73**) have a tajixanthone type skeleton. These compounds were named 14-Hydroxyltajixanthone (**67**), 14-Hydroxyltajixanthone hydrate (**68**), 14-Hydroxyl-15-chlorotajixanthone hydrate (**69**), Epitajixanthone hydrate (**70**), tajixanthone hydrate (**46**), 14-methoxyltajixanthone-25-acetate (**71**), 15-chlorotajixanthone hydrate (**66**), questin (**72**) and carnemycin B (**73**). An anti-agricultural pathogenic fungal assay indicated that compounds **68–70** and **73** displayed significant effects against *Drechslera maydis* with MIC = 25 mg/mL. Moreover, compound **68** exhibited significant effects towards three other fungi, including *Rhizoctonia cerealis*, *F. oxysporum* and *Physalospora piricola*. However, **69** and **71** exhibited an effect against *Rhizoctonia cerealis* with MIC = 25 mg/mL. Compound **46** exhibited a significant effect against *Physalospora piricola* (MIC = 25 mg/mL). With the exception of *Staphylococcus aureus,* compounds **68**–**73** showed significant antibacterial activity against all tested Gram-positive and Gram-negative bacteria, with MIC values ranging from 12.5 to 50 mg/mL, while **68** and **46** showed moderate impacts against *Staphylococcus aureus* with MIC value of 50 mg/mL [60]. 

**Figure 5:**
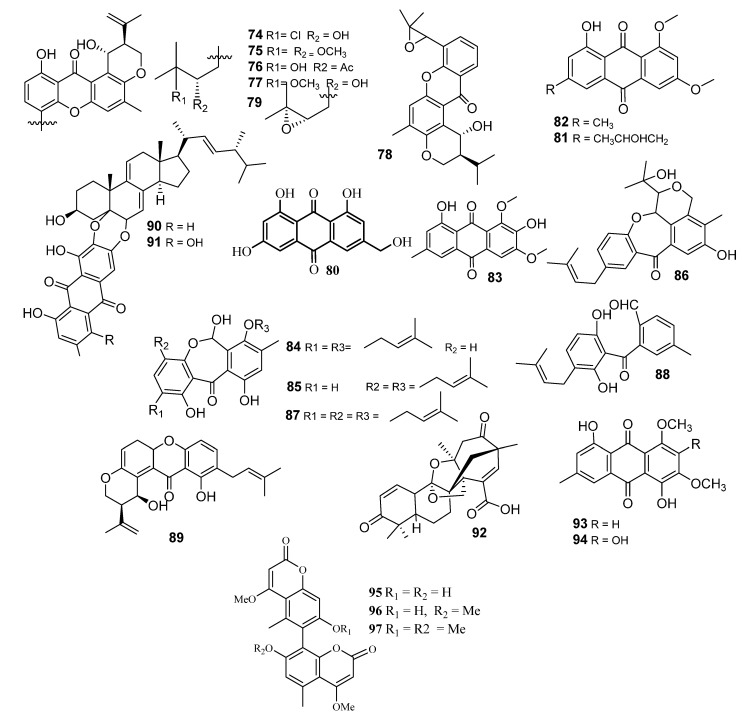
Structures of compounds **74–97**.

Four new prenylxanthones, emerixanthones A–D (**74**–**77**) (Figure 5), along with the six known analogues shamixanthone (**45**), tajixanthone hydrate (**46**), ruguloxanthone A (**53**), ruguloxanthone B (**54**), tajixanthone (**78**) and tajixanthone methonate (**52**), were obtained from the marine fungus *Emericella* sp. SCSIO 05240, which was isolated from deep sea sediment [60]. X-ray crystallographic analysis of ruguloxanthone B (**54**) was performed, aiming at the identification of its stereochemistry. All the isolated compounds were assayed for antibacterial, antifungal and antitumor activities. Compounds **74** and **77** showed weak antibacterial effects against all pathogens. The inhibition zones of **74** and **76** against the six pathogens were in a range between 4 and 6 mm, while that of the positive control ciprofloxacin was in a range between 35 and 40 mm. Compound **77** displayed weak antifungal activity against all agricultural pathogens with inhibition zones of 3–4 mm, while that for the positive control, carbendazim, was 40–45 mm. No effects were observed against the ten tested human tumor cell lines [61].

Chromatographic purification performed on the extract obtained from *Emericella* sp. SCSIO 05240, which was isolated from deep sea sediments [62], resulted in the identification of a new emerixanthone E (**79**) and four known metabolites, orange yellow powder (**80**), citreorosein (**81**) (Figure 5), emodin 6, 8-methyl ether (**82**) and hydroxyemodin 6, 8-methyl ether (**83**), were isolated. The biological properties of those compounds (**79**–**83**) were explored for antifungal, antimicrobial and cytotoxic effect. The isolated compounds **79**–**83** were evaluated for antibacterial, antifungal and cytotoxic activities. Compounds **79** and **80** showed a moderate antibacterial effect (at 50 μg/well) against *Escherichia coli* (ATCC 29922), *Klebsiella pneumonia* (ATCC 13883), *Staphylococcus aureus* (ATCC 29213), *Enterococcus faecalis* (ATCC 29212), *Acinetobacter baumannii* (ATCC 19606) and *Aeromonas hydrophila* (ATCC 7966). The zone of inhibition of **79** and **80** was in the range of 9 and 11 mm against six pathogens which more effective than ciprofloxacin (35 and 40 mm, respectively). None of the compounds showed antifungal and antitumor activity against a panel of ten human tumor cell lines (K-562, A-549, HL-60, Huh7, MCF-7, H-1975, U937, BGC823, Hela and MOLT-4).

Arugosins A–C (**84**–**86**), G and H (**87**, **88**) and epiisoshamixanthone (**89**) were obtained from the endophytic fungal strain *Emericella* sp. [63]. Moderate cytotoxic activities against several tumor cell lines were observed for 84 and 85 [64].

Investigation of the mycelial and broth extracts of *E variecolor* led to the isolation of three new metabolites, including two unreported anthraquinone-steroids, evanthrasterol A and B (**90** and **91**), and an unknown meroterpenoid, emericellic acid (**92**), together with several reported compounds: 14-methoxytajixanthone-25-acetate (**51**), 8-hydroxy-1,3-dimethoxy-6- methylanthraquinone (**93**), 2,8-dihydroxy-1,3-dimethoxy-6-methylanthraquinone (**94**) and tajixanthone hydrate (**46**). Compounds **90** and **91** were evaluated for their in vitro cytotoxicity against five human tumor cell lines. They showed no cytotoxic effects at 10 µg/mL) [65]. 

## 3. Shikimates

### Bicoumarins

Coumarins consist of a benzopyrone nucleus, which could consist of benzo-α-pyrones to benzo-γ-pyrones. They are found in many plants and help defend against herbivores. They also have an appetite-suppressing effect, which may reduce their consumption by animals. They have various biological effects, including bacteriostatic and anti-tumor effects [66]. 

Three rare bicoumarins, desertorins A–C (**95**–**97**) (Figure 5), were reported as the mycelia of *E. desertorum*, which was isolated from Egyptian desert soils. Desertorin A (**95**) was isolated from acetone extract, while desertorins B and C (**96** and **97**) were isolated from acidic dichloromethane extract. These metabolites have unsymmetrical 4-methoxy coumarin dimers. Restriction of rotation around the biphenyl linkage is the reason for their optical activity. The final structure was supported by hydrolytic degradation of the prepared diacetylbiphenyl derivatives [67]. In addition, the structure of desertorins C was confirmed by synthesizing its racemic mixture [67].

## 4. Mevalonates

### 4.1. Meroterpenes 

Meroterpenes are natural products, categorized as polyketide–terpene hybrids with often-potent bioactivities. These compounds have unusual molecular architectures. Thus, they have received high levels of attention from either chemists or pharmacologists, particularly for their biological effects (e.g., anti-inflammatory, cytotoxic and antimicrobial) [68]. 

From the marine *E. variecolor*, varitriol (**98**), varioxirane (**99**) and dihydroterrein (**100**) were isolated [49]. In the NCI’s 60-cell panel, varitriol (**98**) exhibited potent action against renal, CNS and breast cancer cell lines.

**Figure 6:**
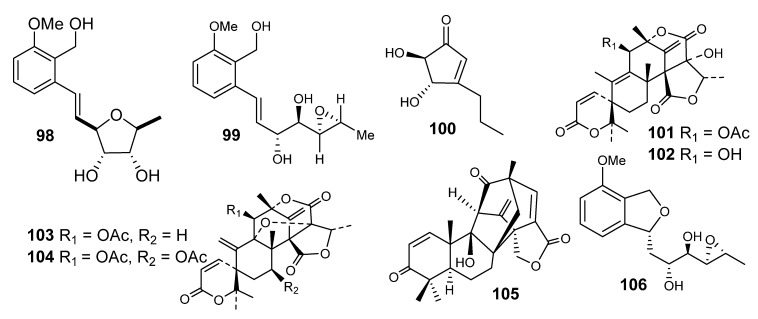
Structures of compounds **98–106.**

Four Austin-like compounds, Austin (**101**), austinol (**102**), dehydroaustin (**103**) and acetoxydehydroaustin (**104**), were isolated from *Emericella* sp. (HK-ZJ) (Figure 6) [69]. These compounds are meroterpenoids and isolated from the *Aspergillus* and *Penicillium* genera. These derivatives have noteworthy levels of toxicities against insects and also block the nicotinic acetylcholine of cockroaches [70]. 

Emervaridione (**105**) and varioxiranediol (**106**) were published from *E. variecolor* (Figure 6) [70].

**Figure 7:**
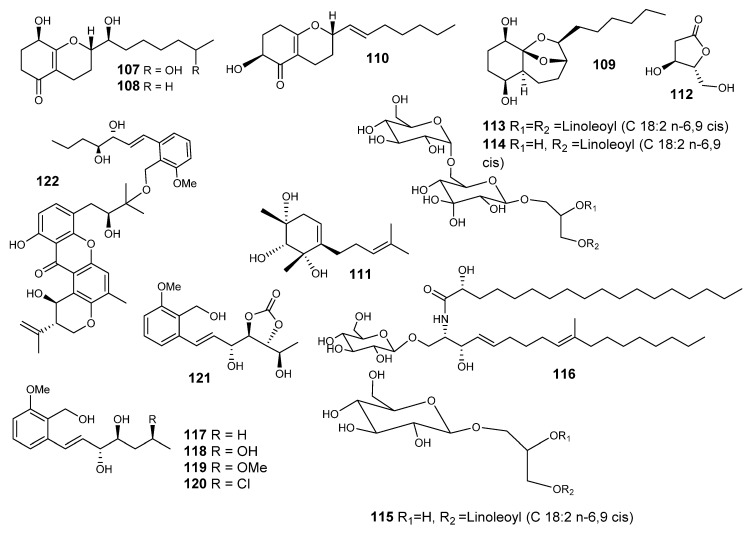
Structures of compounds **107–122.**

Koninginin H (**107**) (Figure 7), a polyketide derivative, was reported from *E. nidulans*, along with koninginin E and A (**108** and **109**), trichodermatide B (**110**), citrantifidiol (**111**), (4*S*,5*R*)-4-hydroxy-5-methylfuran-2-one (**112**), the glycerol derivatives gingerglycolipid B (**113**), (2*S*)-bis[9*Z*,12*Z*]-1-*O*,2-*O*-dilinoleoyl-3-*O*-[α-D-galactopyranosyl-(1″→6)β-D-galactopyranosyl] glycerol (**114**), (2*S*)-bis[9*Z*, 12*Z*]-1-*O*, 2-*O*-dilinoleoyl-3-*O*-β-Dgalactopyranosylglycerol (**115**) and cerebroside flavuside B (**116**), along with the known sterols β-sitosterol glucoside and ergosta-5,7,22-trien-3-ol [71]. Their antifungal effects were assessed towards *Aspergillus (A.) fumigatus*, *Cryptococcus neoformans, C. albicans*, *C. glabrata* and *C. krusei*. Compound **58** exhibited observable antifungal effects against *Cryptococcus neoformans* (IC_50_ = 4.9 μg/mL). The examined compounds showed no effect as antimalarial and antileishmanial assays (Figure 7) [71].

**Figure 8:**
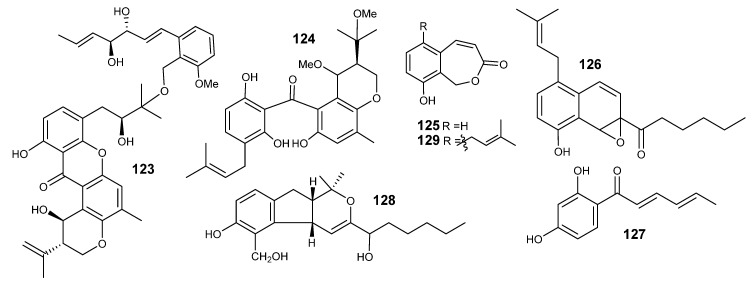
Structures of compounds **123–129.**

Investigation of an *E. variecolor*-derived sponge (*Cinachyrella* sp.) led to the isolation of 7 new polyketides: varioxiranols A−G (**117**−**123**) and a new hybrid polyketide synthase (PKS)-isoprenoid metabolite, 19-*O*-methyl-22-methoxypre-shamixanthone (**124**) (Figure 7 and Figure 8). Varioxiranols F and G were reported as the first metabolites that include a link of xanthone moiety to a benzyl alcohol via an ether bond [72].

*Emericella* sp. IFM57991 afforded several polyketides: xylarinol A (**125**), emericelloxide (**126**) sorbicillin (**127**), aspergillodiol (**128**) and xylarinol C (**129**) (Figure 8) [41]. Compound **128** showed a weak inhibitory effect against *Bacillus subtilis*. Cytotoxicity and antifungal effects were not observed at 50 μM and 250 μg per disk, respectively [41].

**Figure 9:**
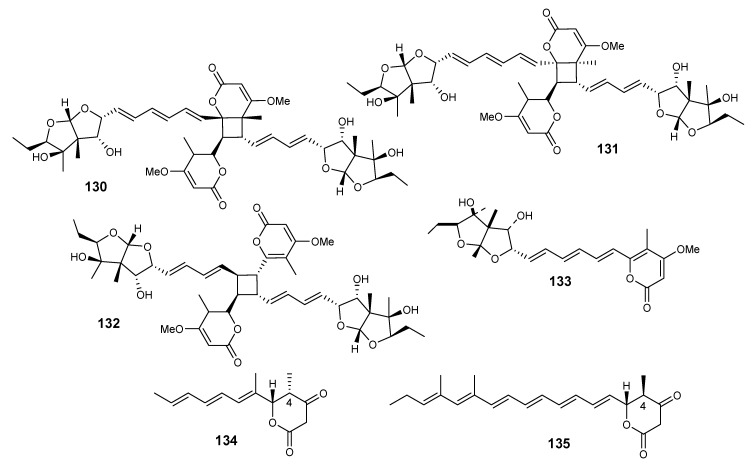
Structures of compounds **130–135.**

Diasteltoxins A−C (**130**−**132**) and asteltoxin (**133**) (Figure 9) were reported from marine *E. variecolor* XSA-07-2 [73]. Compounds **131**−**133** displayed inhibitory effects against H1299 and MCF7, while they displayed observed inhibitions against thioredoxin reductase (TrxR). Two closely asteltoxin-similar compounds **134** and **135** were isolated from *E. variecolor* IFM42010 (an asteltoxin- generating fungal species). Both structures have β-ketolactone moieties; surprisingly they have different stereochemistry at carbon-4 (Figure 9) [74]. The bioassay-directed fractionation of the extract of plant-derived fungus *Emericella* sp. TJ29 yielded three new terpenoidal polyketide hybrid meroterpenoids (emervaridones A−C (**136**−**138**)) and two new polyketides (varioxiranediols A and B (**139** and **140**)), in addition to **99, 105** and **106** [75]. Compound **105** is a unique carbo-skeleton that bears an emervaridone-type carbocyclic moiety. Compounds **136** and **139** exhibited activity against five drug-resistant microbial pathogens with MIC values in the micrograms per milliliter range. Compound **84** showed inhibition effects against extended spectrum β-lactamases (ESBL)-producing *Escherichia coli*, which is comparable to that of the clinically used antibiotic amikacin, with an MIC value of 2 μg/mL. Both **136** and **139** showed low toxicities to mammalian cells [75].

**Figure 10:**
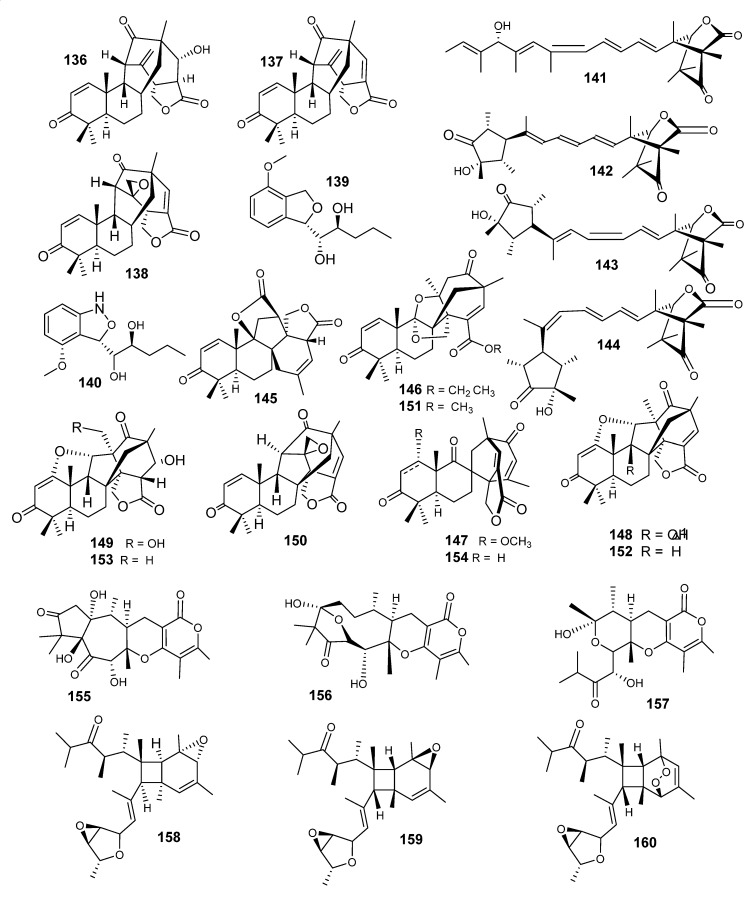
Structures of compounds **136–160.**

An OSMAC application to *Emericella* sp. XL 029 led to the production of 4 unique polyketides, emericelactones A–D (**141**–**144**) (Figure 10). Unique linear pentaene endings with oxabicyclo [2.2.1] heptane moiety are the main features of **141**, while **142**–**144** are isomers including a unique linear triene structure ending in two cyclic moieties of an oxabicyclo [2.2.1] heptane and a cyclopentan-1-one [75]. All the isolated compounds showed moderate antimicrobial activities against 3 fungal stains and two bacterial strains with MIC ranges of 25–50 µg/mL, and compounds (**141**–**144**) were also effective against *P. parasitica* and *B. subtilis* with MIC values in the range of 50–100 µg/mL. It was published that this type of metabolites plays a vital role in protecting its host from parasites, as well as from other predators such as bacteria, fungi and animals [76].

Emeridones A−F (**145**−**150**), 3,5-demethylorsellinic acid-based meroterpenoids, were reported as new natural compounds from *Emericella* sp. TJ29. They were isolated along with 8 known analogs: aspermerodione (**151**), andiconin C (**152**), andiconin B (**153**) (Figure 10), emervaridone C (**134**), spiroaspertrione A (**154**), emervaridione (**105**), emervaridone A (**84**) and emervaridone B (**85**) (Figure 10) [77,78]. Emeridone A (**145**) was the first meroterpenoid featuring a unique rigid 6/6/5/7 tetracyclic carbon ring system with two additional lactone rings. Compounds **140** and **130** have a 2,6-dioxabicyclo[2.2.1] heptane and a spiro[bicyclo[3.2.2]nonane-2,1′-cyclohexane]moiety, respectively. Compounds **146**, **148** and **150** displayed moderate cytotoxic effects (IC_50_ ranges between 8.19–18.80 *μ*M) [77,78]. Recently, three new polyoxygenated a-pyrones merosesquiterpenes, emerones A–C (**155**–**157**), were isolated from E. sp. XL029. Compounds **155**–**157** are characterized by a 5/7 bicyclic skeleton, a rare 10-membered ring and a norsesquiterpene structure, respectively [79]. The antimicrobial activity of these metabolites showed weak to moderate profiles [79]. The extract of *E. nidulans* afforded a number of polyketides, emeriones A–C (**158**–**160**), decorated with huge methyl functions. The compounds are characterized by bicycle[4.2.0]octane and 3,6-dioxabicyclo[3.1.0]hexane skeletons [80]. Emerione A (**158**) showed a weak anti-inflammatory effect [80].

### 4.2. Sesquiterpenes 

**Figure 11:**
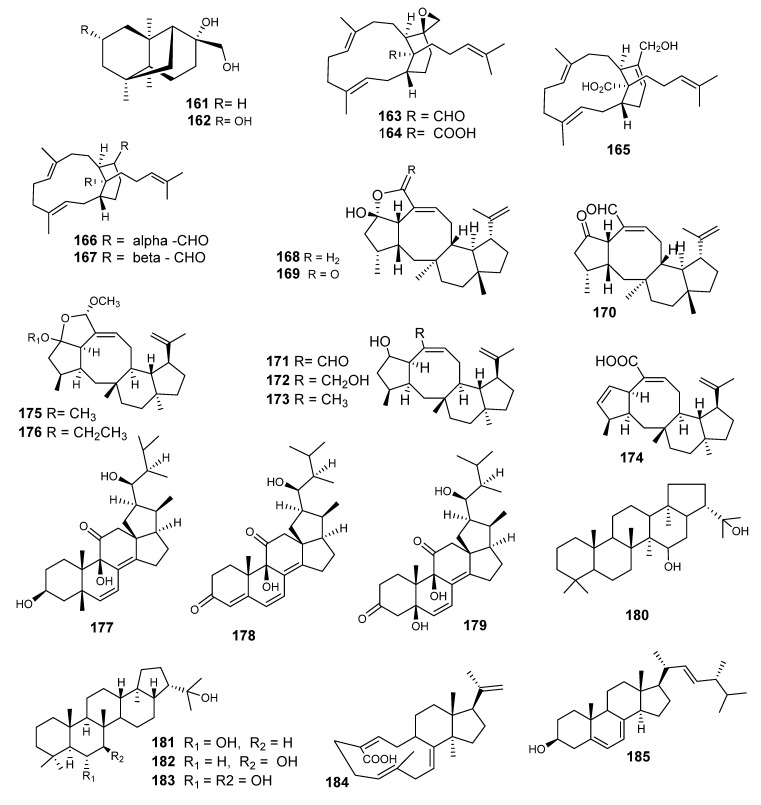
Structures of compounds **161–185.**

Two unprecedented sesquiterpenes with a tricycle[4,4,2,1]hendecane carbon skeleton, Emericellins A (**161**) and B (**162**) (Figure 11), were reported from the endophytic fungus *Emericella* sp. XL 029 [81]. Compounds **161** and **162** displayed moderate activities against three fungal strains, as well as three bacterial strains (*Bacillus subtilis, B. cereus* and *E. coli*) with MIC values of 25–50 μg/mL.

### 4.3. Sesterterpenes

Sesterterpenes are a group of pentaprenyl terpenoidal derivatives whose structures are derivable from geranyl farnesyl diphosphate. They are a small group of terpenoids obtained from different sources: terrestrial fungi, lichens, higher plants, insects and sponges. Sesterterpenoids exhibit diverse biological effects such as anti-inflammatory, cytotoxicity, anti-tumor and antimicrobial activities [82].

Emericellenes A−E (**163–167**), unique sesterterpenes (Figure 11), were reported from the *Emericella* sp. AST0036 isolated from the leaves of *Astragalus lentiginosus* [83]. From a biosynthesis point of view, metabolites **163–167** may be derived from geranylfarnesyl biphosphate; moreover, they represent an unprecedented class of sesterterpenes bearing an emericellane-type bicyclic system. **163**–**167** are found to be non-toxic to a set of several cell lines. 

Variecolol (**168**) and variecolactone (**169**), both sesterterpenes, were published from *E purpurea*, along with **170** (Figure 11). These compounds were also isolated from *E aurantiobrunnea.* They were also reported from *E. variecolor* as an angiotensin II receptor binding inhibitor [84,85]. Variecolin is classified under ophiobolin natural products. Emericolin A–D (**171–174**), two further variecolin analogues, and variecoacetals A (**175**) and B (**176**), were reported from *E aurantiobrunnea* [86,87]. 

### 4.4. Steroids

Three new 18, 22-cycloergostane-type steroids identified as 11-oxo-18,22-cycloergosta -6,8(14)-diene-3β,5β,9β,23S-tetrol (Mer-NF8054X, **177**), emesterone A (3,11-dioxo-18,22-cycloergosta-6,8(14)diene-5β,9β,23S-triol, **178**) and emesterone B (3,ll-dioxo-18,22-cycloergosta-4,6,8 (14)-triene- 9β,23S-diol**, 179**), were elucidated along with the known steroid dustatin (**180**) [41], all identified from *E heterothallica* [59,88,89,90]. Emesterones A (**178**) and B (**179**) were the second reported examples of 18, 22-cyclosterols isolated from fungi. The antifungal activity of emesterone B (**179**) against *A. fumigatus* was less potent than that of Mer-NF8054X (**117**). All of the stereochemistry remaining in **178** and **179** is the same as in ergosterol and Mer-NF8054X (**177**), which might be a precursor of **178** and **179**. 

Zeorin (**181**), hopane-7β,22-diol (**182**) and hopane-6α:,7β,22-triol (**183**) are triterpenes. Zeorin was reported as a typical lichen metabolite. Hopane-6α, 7β, 22-triol has also been isolated from lichens, while Hopane-7 β,22-diol has not been found previously in lichens, but its acetate has been isolated for the first time as a hopane-type triterpene (Figure 11) without an oxygen function at C-3 (it was isolated from *Aspergillus* sp. but was identified as dustanin (hopane- l5α,22-diol)). It is also found in dust in the air as well as in an entomogenous fungus, *Aschersonia aleyrodis* [91]. Investigation of an *E. variecolor* organic extract led to the isolation of stellatic acid (**184**) and ergosterol (**185**) [91,92].

## 5. Amino Acids Derivatives

### 5.1. Alkaloids

#### 5.1.1. Indole-Derivatives

Paxilline (**186**) was first published from *Penicillium paxili*, which was isolated from insect-damaged pecans. Paxilline was also isolated from *Acremonium lorii* as a biosynthetic precursor of loritrem B, which induces a neurotic syndrome affecting sheep. On theses bases, a further investigation was reported, which was interested in detecting the paxilline in 19 *Emericella* species, and was found in *E. desertorum*, *E. foveolata*, and *E. striata* [93]. A derivative of paxilline (l’-0- acetylpaxilline, **187**), was isolated from the mycelium of *E. striata*. Paxilline induced tremors in cockerels and mice after oral administration (25 mg/kg). Deep investigation indicated that it had an effect on the electromyographic (EMG) characteristics of smooth muscle in the reticulorumen of conscious sheep [93].

**Figure 12:**
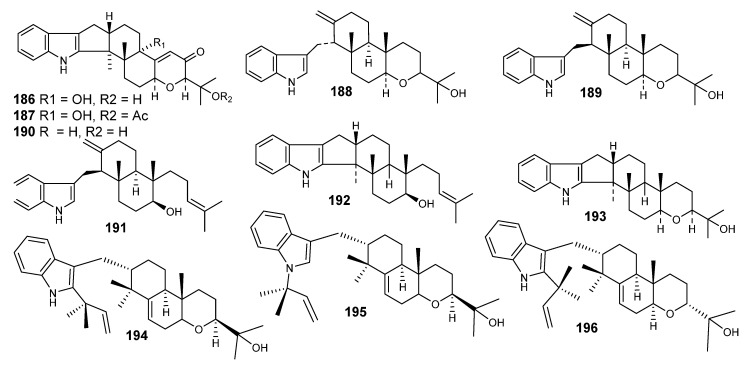
Structures of compounds **186–196.**

Three more derivatives, namely emindoles DA (**188**), DB (**189**) and SA (**190**) (Figure 12), were identified from *E. desertorum* and *E. striata*. Similar compounds, designated dehydroxypaxilline **(188),** emindole SB **(192**) and paspaline **(193),** were also isolated [94,95,96,97,98,99]. 

Emindoles PA–PC (**194–196**) are indoloditerpenes, identified from the mycelium of *E. purpurea* [100]. The common feature among these metabolites is the presence of 1,1-dimethyl-2-propenyl residues at C-2 or N-1 in the indole moiety. Those metabolites are entirely biosynthesized from geranylgeraniol and a tryptophan biosynthetic pathway [100,101].

#### 5.1.2. Isoindolones

**Figure 13:**
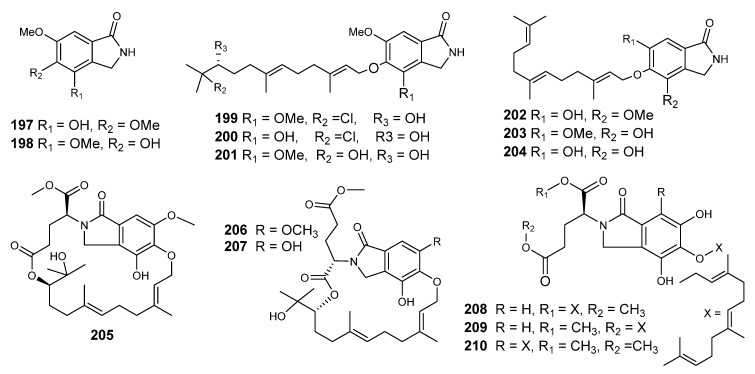
Structures of compounds **197–210**.

*Emericella* sp. (HK-ZJ) is an endophytic fungus that was isolated from the mangrove plant *Aegiceras corniculatum.* Eight isoindolones derivatives, emerimidine A and B (**197,198**) and emeriphenolicins A–F (**199–204**), were isolated (Figure 13) [68]. Compounds **197** and **198** displayed mild inhibitory effects on the replication of the influenza virus H1N1 in MDCK cells by employing a cytopathic effect (CPE) inhibition assay, with IC_50_ values of 42.07 µg/mL and 62.05 µg/mL, respectively [68].

Six isoindoline-containing meroterpenes, emericellolides A−C (**205**−**207**) and emeriphenolicins E−G (**208**−**210**), were obtained from a culture of the endophytic fungus *E nidulans* HDN12-249. This fungus was isolated from the leaves of *Tamarix chinensis*, which was collected from Laizahau Bay [102]. Compounds **204**−**207** have a unique macrolide carbo-skeleton of an unusual L-glutamate fragment, an isoindolone and a sesquiterpene moiety, while the structures of emeriphenolicins E−G (**208**−**210**) had two farnesyl groups attached to one isoindolone unit (Figure 13), which are rare isoindolone-derived meroterpenoids. The cytotoxic effects of compounds (**208**−**210**) were assessed against HeLa, A549 and HCT-116, human cancer cell lines, with adriamycin as a positive control. Compound **205** showed cytotoxic effects with IC_50_ values of 4.7, 12.04 and 33.05 μM, respectively. However, the rest of the compounds were not active (IC_50_ > 50 μM) [102].

#### 5.1.3. Piperazines

Piperazines are an organic substance that consists of a six-membered ring with two nitrogen atoms at opposite positions in the ring. They are a big class of chemical compounds with different pharmacological properties, which contains a piperazine group [103]. Epidithiodioxopiperazine is a group of alkaloids with an amazing array of molecular structure and potent bio-activities [104,105,106]. 

A macrocyclic epidithiodioxopiperazine, emestrin (**211**), was reported from *E striata* (80-NE-22). This strain was isolated from the seeds of *Cuminum cymium*, which were collected from Nepal (Figure 15) [104,105]. The chemical structure was obtained by interpretation of the spectral data of ^1^H-, ^13^C-NMR and MS. An X-ray was performed after obtaining crystals of emestrin from methanol, which were grown in an acetone-methanol solution as prisms. The planarity between the two aromatic rings was not so good, because of the formation of the 15-member ring [106]. Further investigation of a filtrate of the same culture led to the identification of violaceic acid. Emestrin (**211**) inhibited the growth of *Gibberella zeae* and *Penicillium expansum* (1.0 µg per disc) with 12 and 10 mm inhibition circle diameters, respectively. The MIC values of **80** against *G. zeae* and *P. expansum* were 10 and 2.5 µg/mL, respectively [105,106].

Emethallicin A (**212**) (epidithiodioxopiperazioid) was isolated from *E heterothallica* (mating type A), along with ergosterol. It is characterized by the presence of mandelic and phenylacetic diester moieties [107]. Emethallicin A (**212**) had a potent inhibitory effect on either histamine release or 5-lipoxygenase (IC50 = 3.0 × 10-8 and 1.7 × 10-6 M, respectively) [107]. 

Emeheterone (**213**) was the first pyrazinone derivative that has been derived from two aromatic amino acid moieties. It was isolated from the dichloromethane extract of *E heterothallica*, strain ATCC 16824, along with dithiosilvatin (**214**) and a dihydroisocoumarin (Stellatin, **215**) [108]. 

**Figure 14:**
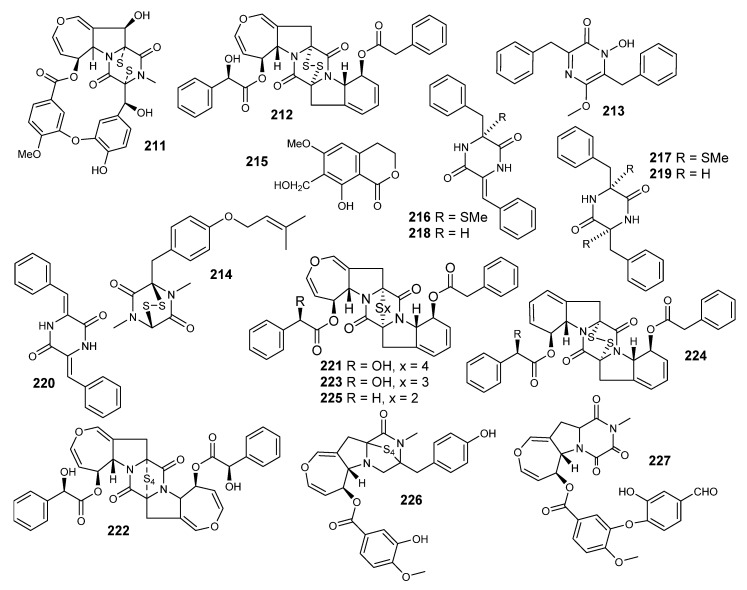
Structures of compounds **211–227.**

Emethacins A and B (**216**–**217**) (Figure 14) are sulfur-containing dioxopiperazines, which were isolated from a heterothallic fungus, *E heterothallica* [109]. In addition, there were three dioxopiperazine derivatives: (3S.6Z)-3-Benzyl-6-benzy1idene-2,5-dioxopiperazine (**218**), (3S,6S)-3.6-dibenzyl-2,5-dioxopiperazine (**219**) and (3Z,6Z)-3,6-Dibenzylidene-3,5-dioxopiperine (**220**). Emethacin B was identical to (3*R*,6*R*)-3,6-dibenzyl-3,6-bis(methylthio)-2,5-dioxopiperazine (**219**), previously reported from *A. terrus*. Emethacin A (**216**) represents the first natural mono- (methylthio)dioxopiperazine skeleton. It is interesting that there are mono and bis (methylthio) dioxopiperazines [110]. 

Emethallicins B, C, and D (**221**–**223**), along with emethallicin A (**204**), were published from the *E. heterothallica* [110]. Compounds **221** and **222** are epitetrathiodioxopiperazines, while emethallicin D (**223**) is an epitrithiodioxopiperazinoid (Figure 14). It is worth mentioning that a large amount of the disulfides **(216**, **217)** and the trisulfide emethallicin D (**223**) was isolated from the other mating-type strain, along with a small amount of the disulfide. Emethallicins (**221**–**223**) exhibited a potent inhibitory effect on compound 48/80-induced histamine release from mast cells, and are also 5-lipoxygenase inhibitors, like emethallicin A (**212**). The IC_50_ values for inhibition of histamine release were evaluated to be in the range of 3.0 × 10-8 - I.O × 10-6 M for emethallicins A, B and C, emethallicin A monoacetate, emethallicin B diacetate and emethallicin D monoacetate, whereas those for the inhibition of 5-lipoxygenase were determined as 1.7 × 10^-6^, 1.3 × 10^-6^ and 2.6 × 10^-6^ M for **221**, **212** and **222**, respectively. Two more emethallicins were isolated from *E*. *heterothallica* E and F (**224** and **225**). Like other emethallicins, they were found to inhibit the histamine release [111]. A new cytotoxic epitetrathiodioxopiperizine derivative, Secoemestrin D (**226**), was isolated from *Emericella* sp. AST 0036. Compound **226** showed significant activity against 6 selective cancer cell lines related to normal human fibroblast cells [82]. Another emestrin derivative, dethiosecoemestrin (**227**) was isolated from the dichloromethane extract of *E. striata*. Compound **227** showed good antibacterial activity against *E. coli* (10µg/disc) [112].

### 5.2. Cyclic Peptides and Depsipeptides 

Peptides are a chain of amino acids linked by amide bonds. They include, but are not limited to, dipeptides, tripeptides and tetrapeptides. A polypeptide is a long, continuous, and unbranched peptide chain [113]. Depsipeptides are basically peptides, where some of the cyclic peptide amide functions are replaced by the corresponding esters [113]. Cyclic peptides are mainly isolated from terrestrial microorganisms and marine organisms [112]. 

**Figure 15:**
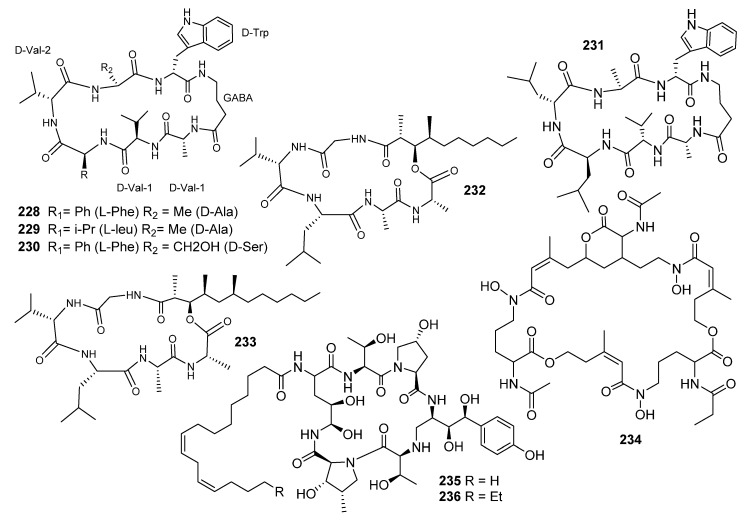
Structures of compounds **228–236.**

Unguisins A–C (**228**–**230**) were reported as the first cyclic heptapeptides of the gamma aminobutyric acid (GABA) skeleton, isolated from the marine-derived fungus *E. unguis,* which was isolated from a Venezuelan cannonball jellyfish, *Stomolopus meliagris* [114,115]. **228** and **229** (Figure 15) displayed moderate effects against *Staphylococcus aureus*, but no activity against *Vibrio parahaemolyticus*. Furthermore, the exogenous addition of L-leucine to the culture resulted in the production of unguisin D (**231**), which was detected by ion trap mass chromatography (LC-QIT-MS) [116]. Cyclic peptides are bio-synthesized through either ribosomal or nonribosomal mechanisms. The high content of D-amino acids in unguisins A and B strongly supports the indication of a nonribosomal origin for these metabolites. Fungi utilize GABA as a carbon and nitrogen source, and it is associated with some of the major features of the cell cycle, including sporulation, differentiation and development. The presence of GABA in (**228–230**) induces an enhanced conformational mobility relative to the cyclic peptides derived solely from R-amino acids. Such conformation-biological activity dependence strengthens the occurrence of GABA in **228**- **230** [114,115,116]. 

Unguisin C (**230**) allowed the replacement of D-alanine with L-serine, leading to a peptide with more hydrophilicity, based on the oxidation of the methyl group in alanine to produce serine [116]. Unguisines **228**–**230** are the first naturally occurring compounds incorporating GABA in a cyclic peptide structure. 

Emericellamides A and B (**232** and **233**) reported from *Emericella* sp., when cultured with the actinomycete *salinispora arenicola* [117]. Emericellamides A and B (**232**–**233**) (Figure 15) showed moderate antibacterial activities towards methicillin-resistant *Staphylococcus aureus* with MIC (3.8 and 6.0 µM, respectively) [117,118,119]. 

Incorporation of the cyanobacterium *Scytomena ocellatum*, with added elicitors (e.g., fungal wall polysaccharides), led to increasing the production of tolytoxin [120]. The marine fungus *Phomopsis asparagi* cultured in the presence of the spongal product jasplakinolide, which redirected its synthetic pathway to yield chaetoglobosins. Mixed cultivation of the marine-derived fungus *Libertella* sp. with a marine strain with α-proteobacterium, closely related to *Thalassospira lucentensis*, yielded new cytotoxic diterpenoids. Desferritriacetylfusigen (**234**) was one of the natural group of a cyclic triester of the three molecules of N-acetyl-N-(cis-5-hydroxy-3-methylpent-2-enoyl)-N-hydroxy-L-ornithine, which are known for their antibiotic effects. These compounds are detected in most fungal species [121,122]. Echinocandin E (235) was identified from E. quadrilineata and was reported for the first time as producing echinocandin B (**236**) [123]. 

Based on the scientific literature published on the genus *Emericella* and some other fungal species [124,125,126,127,128], fungal metabolites could replace some conventional shelf drugs that are common in the market with extremely few drawbacks, for example cordycepin (a nucleoside obtained from *Cordyceps militaris*) replaces glyophosate and benzoic acid. Cordycepin showed a maximum inhibition on the germination and growth of *Raphanus sativus* (radish) (IC_50_ = 0.052–0.078 mg/mL).

## 6. Conclusions and Future Prospective

In terms of the genus *Emericella*, a careful review of the major carbon skeletons revealed the involvement of one or several biosynthetic pathways involved in the production of diverse metabolites. It proved to be a productive genus, as 236 metabolites have been isolated and classified into polyketides (azaphilones, cyclopentenone pigments, dicyanides, furan derivatives, phenolic ethers, xanthones and anthraquinones); Shikimate derivatives (bicoumarins); Mevalonate derivatives (meroterpenes, sesquiterpenes, sesterterpenes and steroids) and amino acids derivatives (alkaloids (indole-derivatives, isoindolones and piperazine) and peptides (depsipeptides)). 

Among the 236 secondary metabolites isolated, almost 30% were not examined for their biological impacts and 43% showed weak to absent activities in the conducted bioassay (mainly antimicrobial and cytotoxicity assays). However, there are several bright and motivating examples encouraging more bio-investigation of the *Emericella* isolates. Stromemycin (a xanthone derivative) was patented for its metalloproteinase-inhibiting effect. Falconensins E, K and M displayed inhibitory effects against the enhancement of ear edema in mice, which was comparable with indomethacin. Tajixanthone hydrate and 14-methoxy tajixanthone-25-acetate have been shown to be active as doxorubicin toward KATO3 and BT474. Ruguloxanthone A and 15-chlorotajixanthone hydrate displayed a potent inhibition of the calmodulin-sensitive cAMP phosphodiesterase (PDE1). This effect is comparable with that of chlorpromazine, a well-known Calmodulin (CaM) inhibitor. As far as reported, neither clinical trials nor randomized control trials (RCTs) have been conducted on these compounds.

It is interesting to note the high conjugation system that was observed amongst the yellow-colored cyclopentenone derivatives. This is an important observation, which aims at encouraging researchers to recall these compounds as antioxidants and anti-tumour. Moreover, indoloterpenes are well-known biologically active compounds (chiefly isolated from plant families such as Apocynaceae) formed from tryptophan and terpenoid substrates. The presence of tryptophan derivatives (e.g., Emindoles) could lead to the discovery of an agent that improves cognition and reduces insomnia and depression. It is also noteworthy that nine peptides were reported as having cyclic heptapeptides containing GABA in the ring, which opens the door to the discovery of new antifungal agents. Other carbon skeletons such as phenols, meroterpenes and ethers were published from the genus *Emericella*, and there is still potential to find new carbon skeletons and/or biological rules, proving the value of analyzing fungi to promote drug discovery.
